# Genetic interaction network of the *Saccharomyces cerevisiae *type 1 phosphatase Glc7

**DOI:** 10.1186/1471-2164-9-336

**Published:** 2008-07-15

**Authors:** Michael R Logan, Thao Nguyen, Nicolas Szapiel, James Knockleby, Hanting Por, Megan Zadworny, Michael Neszt, Paul Harrison, Howard Bussey, Craig A Mandato, Jackie Vogel, Guillaume Lesage

**Affiliations:** 1 Department of Biology, McGill University, Montreal (QC), Canada; 2 Anatomy and Cell Biology, McGill University Montreal (QC), Canada; 3 Developmental Biology Research Initiative, McGill University, Montreal (QC), Canada; 4 Cell Imaging and Analysis Network (CIAN), McGill University, Montreal (QC), Canada

## Abstract

**Background:**

Protein kinases and phosphatases regulate protein phosphorylation, a critical means of modulating protein function, stability and localization. The identification of functional networks for protein phosphatases has been slow due to their redundant nature and the lack of large-scale analyses. We hypothesized that a genome-scale analysis of genetic interactions using the Synthetic Genetic Array could reveal protein phosphatase functional networks. We apply this approach to the conserved type 1 protein phosphatase Glc7, which regulates numerous cellular processes in budding yeast.

**Results:**

We created a novel *glc7 *catalytic mutant (*glc7-E101Q*). Phenotypic analysis indicates that this novel allele exhibits slow growth and defects in glucose metabolism but normal cell cycle progression and chromosome segregation. This suggests that *glc7-E101Q *is a hypomorphic *glc7 *mutant. Synthetic Genetic Array analysis of *glc7-E101Q *revealed a broad network of 245 synthetic sick/lethal interactions reflecting that many processes are required when Glc7 function is compromised such as histone modification, chromosome segregation and cytokinesis, nutrient sensing and DNA damage. In addition, mitochondrial activity and inheritance and lipid metabolism were identified as new processes involved in buffering Glc7 function. An interaction network among 95 genes genetically interacting with *GLC7 *was constructed by integration of genetic and physical interaction data. The obtained network has a modular architecture, and the interconnection among the modules reflects the cooperation of the processes buffering Glc7 function.

**Conclusion:**

We found 245 genes required for the normal growth of the *glc7-E101Q *mutant. Functional grouping of these genes and analysis of their physical and genetic interaction patterns bring new information on Glc7-regulated processes.

## Background

Regulation of phosphorylation state is a mechanism for controlling the function, localization and stability of proteins *in vivo *and is critical for the regulation of essential processes such as polarity and morphogenesis, chromosome segregation, cytokinesis, and cell cycle control [1-4]. Together, protein kinases (which mediate phosphorylation) and protein phosphatases (PPases) (which mediate de-phosphorylation) provide precise temporal and spatial regulation of their target substrates. In the budding yeast *Saccharomyces cerevisiae*, the dynamic localization of PPases suggests that an extensive cross talk between these processes is critical for the proper execution of cell division [2,5]. However, many of the substrates and regulatory proteins that participate in this cross talk remain unidentified. Insight into PPase function has lagged significantly in comparison to kinases since PPases: (1) frequently require regulatory proteins that determine their targeting to particular substrates, cellular location or process and (2) often exhibit considerable functional redundancy. In budding yeast, for example, ~90% of the known 32 PPases are non-essential [6].

Study of genetic interaction networks is a powerful means to get insight into gene function, and several high throughput methods were developed during the last decade to generate genome-wide maps of genetic interactions [7-13]. However, such maps are still missing for yeast PPases. To date investigations of PPase function in budding yeast have utilized mutations outside the catalytic site [5,14-16] or probed genetic interactions using single or double knockout PPase mutations [6]. These approaches present constraints for large-scale analysis of PPase function since: (1) mutations outside the catalytic domain generally affect the formation of a specific class of holoenzyme or the ability to interact with co-factor(s) required for the proper targeting of the PPase to a subset of substrates [17-19], and (2) the use of knockout alleles, in addition to precluding the analysis of the essential PPases, may lead to promiscuous and/or low-affinity interaction of regulatory subunits with PPases of the same class. For these reasons, we designed a strategy based on a hypomorphic catalytic PPase mutation that uses the Synthetic Genetic Array (SGA). This method generates a large number of double mutant combinations, and has been successfully used to identify genetic interaction networks in yeast in a wide range of cellular processes [12,13,20,21].

In the budding yeast *Saccharomyces cerevisiae*, 32 genes encode predicted or demonstrated catalytic subunits of PPases. Only two of these PPases are clearly essential: the PP1-type PPase, Glc7 (**gl**y**c**ogen deficient) [1,22], and Cdc14 (**c**ell **d**ivision **c**ycle), a dual-specificity phosphatase that regulates mitotic/meiotic exit [23]. We selected Glc7 for our analyses since a framework of genetic and protein interactions exists from previous studies of conditional *glc7 *mutants, but a comprehensive large-scale genetic analysis had not yet been performed. Glc7 critically regulates numerous processes such as glucose and glycogen metabolism, sporulation, chromosome segregation, meiosis, mRNA transport, transcription, and amino acid biosynthesis [1,3,15,17,22,24,25]. A search in the BioGRID database (version 2.0.41, June 1, 2008 [26]) returned a list of 114 proteins reported to physically interact with Glc7. Some of these proteins are known substrates (Cbf2, Fin1, Red1, Gsy2), while others are thought to be regulatory subunits (Gac1, Reg1, Reg2, Ref2, Sip5, Glc8, Bud14, Bni4, Sds22). However, the functional significance of the majority of these interactions remains unclear. In contrast, only 30 genes have been reported to show genetic interactions with various *glc7 *mutants. The vast majority of these genetic interactions are dosage dependent and only 7 genes (*BUD14*, *DAM1*, *DOC1*, *GLC8*, *RHR2*,*SET1 *and *SLT2*) have been reported to show synthetic lethal/sick interaction with different conditional *glc7 *alleles. The functional clustering of genetic interactions of conditional *glc7 *alleles suggests the majority of these mutations compromise specific aspects of Glc7 function. As such, these alleles are highly suited for cell biology. However an unbiased large-scale analysis revealing a comprehensive picture of Glc7 signaling *in vivo *requires a novel *glc7 *allele affecting many functions of Glc7 at a time.

In an effort to further define the signaling network for Glc7, we created a novel *glc7 *catalytic mutant (*glc7-E101Q*) suitable for SGA. The *glc7-E101Q *mutant exhibited slow growth and impaired glycogen accumulation on glucose, but no specific delay in the cell cycle. The impaired growth and defects in glycogen synthesis of the *glc7-E101Q *mutant were rescued by introduction of a single copy of wild type *GLC7*, indicating that *glc7-E101Q *is a recessive, hypomorphic allele. SGA analysis revealed a broad network of 245 synthetic sick/lethal (SSL) interactions that encompassed all known Glc7-regulated processes, and suggested additional previously unknown functions for Glc7.

## Results

### Creation of a catalytic-deficient *GLC7 *mutant, *glc7-E101Q*

**Figure 1:**
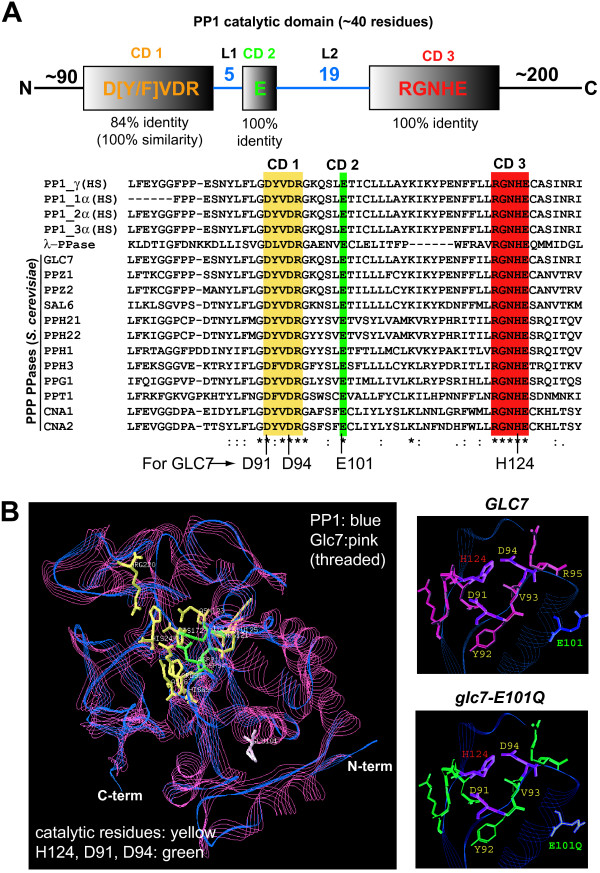
**Structure of PP1 catalytic motif and the *glc7-E101Q *mutant**. (A) PP1 protein phosphatases have highly conserved catalytic domains, consisting of residues acting as an acidic catalyst for phosphotransfer (CD1 and CD2) from the substrate and a phospho acceptor hisidine residue (CD3). Residues in the catalytic pocket are highly conserved between human (PP1), yeast PPP-type PPases (Glc7 and others) and the bacterial PPase, λ-PPASE. (B) *Left panel*: The Glc7 catalytic pocket (pink) was modeled using the tertiary structure of human PP1C-γ (PDB Model 1jk7A; blue). *Right panels*: The E101Q mutation is predicted to inhibit the binding of a metal cation required to accelerate phospho-transfer to H124, but to not alter the shape/size of the catalytic pocket relative to wild type Glc7.

Glc7 is the sole essential member of the PPP family of PPases in budding yeast and members of this family share a conserved catalytic motif. Residues in the Glc7 catalytic domain (CD1-3) are evolutionarily conserved with human PP1 orthologs and the PP1-like bacterial λ-PPASE (Figure 1A). Given the high degree of conservation in the catalytic residues, Glc7 is expected to have catalytic characteristics similar to those of previously characterized PP1-type PPases. Phosphate hydrolysis by PP1 occurs via a onestep reaction and is dependent on metal ion catalysts (Mn^2+ ^and Fe^2+^). This hydrolysis reaction (Additional File 1) is dependent on a phosphoesterase motif DXH(X)_n_G**D**XX**D**(X)_n_GN**H**D/E (where n = ~25 amino acids) whose active residues are a general acid catalyst (H124 in Glc7) and the carboxyl oxygen of two invariant aspartic acids (D91 and D94 in Glc7) [27]. Similar to human PP1 and Glc7, λ-PPASE (a bacteriophage ortholog of PP1) shares this conserved catalytic domain (Figure 1A) and has been previously examined in structure-function analyses of the active residues in the catalytic pocket. Mutation of either D49 or D52 (D92 and D95 in PP1C-γ; D91 and D94 in Glc7, respectively) to a non-reactive asparagine residue (D>N) resulted in a catalytically dead enzyme in *in vitro *PPase assays. However, mutation of E59 (E>Q; E102 in PP1C and E101 in Glc7) reduced the catalytic activity of λ-PPASE ~8 fold and impaired Mn^2+ ^recruitment, but did not significantly alter its substrate binding [28]. An E>Q substitution in the equivalent residue of Glc7 (E101Q) is therefore predicted to reduce catalytic activity by perturbing the recruitment of the metal co-factor that enhances phospho-transfer. Using human PP1C-γ as a template, we found that the conserved residues of Glc7 in the CD1-3 regions form a tertiary structure very similar to human PP1. Furthermore, analysis of the 3-D model of the E101Q mutation in Glc7 indicated that the overall shape and size of the catalytic pocket was not significantly changed (Figure 1B). We predicted that reduction of Glc7 catalytic activity *in vivo*, as a result of the E101Q mutation, would result in a recessive, hypomorphic *glc7 *allele suitable for an unbiased SGA analysis.

### *glc7-E101Q *mutant exhibits slow growth but no appreciable cell cycle delay or defect in chromosome segregation

**Figure 2:**
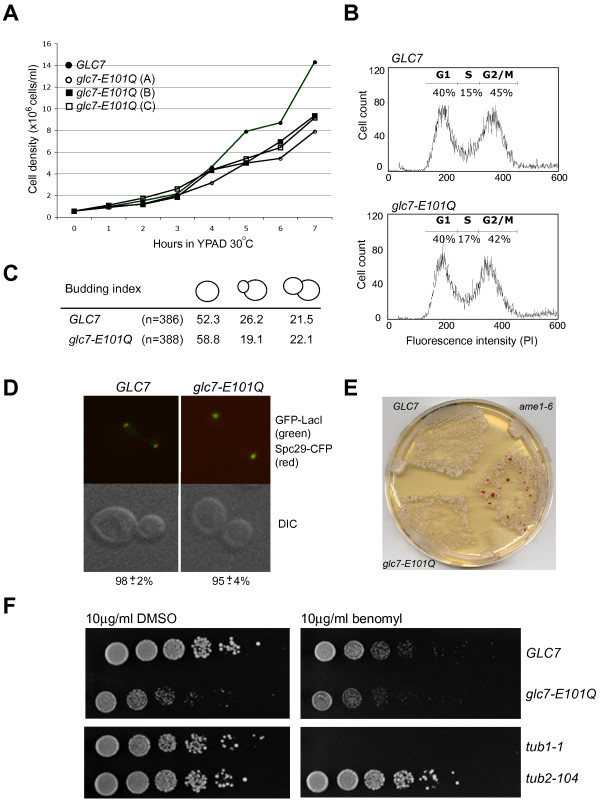
***glc7-E101Q *mutant exhibits slow growth but no appreciable delay in the cell cycle or chromosome segregation defect**. (A) The growth of wild type (*GLC7*) vs *glc7-E101Q *strains in rich medium (YPAD) was determined from early log-phase cultures. (B) FACs analysis for DNA content (propidium iodide staining) of log-phase wild type and *glc7-E101Q *cells. Cell counts are shown plotted against fluorescence intensity (PI). (C) Budding index of wild type and *glc7-E101Q *mutant strains. (D) Fluorescence microscopy of anaphase cells (mitotic spindle >2.5 μm) wild type and *glc7-E101Q *cells expressing a chromosome [GFP-LacI (green) and centromeric (CEN15) repeats of the lactose operon (lacO)] and spindle pole body (SPB) [Spc29-CFP (red)] markers. The percentage of cells with chromosomes localized to both SPBs is indicated at the bottom (n = 3, 300 cells/experiment). (E) Test for chromosome segregation using a sectoring assay. Wild type *GLC7*, *glc7-E101Q *and *ame1-6 *strains lacking a functional *ADE2 *gene (*ade2Δ-101*) and transformed with a Circle III/*SUP11 *were plated on to medium lacking adenine (YPD) and scored for appearance of red sectors after 4-day incubation. (F) Spotting assay of *glc7-E101Q *vs. wild type on DMSO control or benomyl plates (5-fold serial dilutions of 1.0 × 10^6 ^cells/ml; 5 μl/spot). Benomyl-sensitive (*tub1-1*) and resistant (*tub2-104*) strains were plated as controls.

The introduction of a *glc7-D91N *mutation into diploid cells resulted in inviable haploid progeny by tetrad dissection (data not shown). This finding is consistent with the previous observation that this mutation results in a catalytically dead form of the enzyme *in vivo *[28]. In contrast, haploid *glc7-E101Q *colonies isolated by tetrad dissection were viable but consistently small compared to wild type (*GLC7*) when grown on rich medium (YPAD) (data not shown). Analysis of the growth curve for three haploid isolates of *glc7-E101Q *indicated impaired kinetics compared to wild type cells (Figure 2A). To determine whether slow growth was due to a delay in cell cycle progression, mutant and wild type cells from log phase cultures were harvested, stained with propidium iodide and DNA content measured by FACS analysis. As shown in Figure 2B, FACS analysis revealed that proportion of G1, S, or M-phase cells in *glc7-E101Q *cultures was not statistically different from that of wild type cells. This result correlated with the observation that proportion of unbudded, small-budded and large-budded cells was similar between wild type and *glc7-E101Q *strains (Figure 2C).

Studies using conditional *glc7 *mutants have demonstrated a role for Glc7 in the attachment of spindle microtubules to kinetochores during metaphase and chromosome segregation [1,29-33]. Therefore, we next examined *glc7-E101Q *cells for chromosome segregation defects and sensitivity to the microtubule-destabilizing agent, benomyl. To examine bi-orientation of sister chromatids, anaphase cells (mitotic spindle>2.5 μm) expressing GFP-LacI and centromeric repeats (CEN15) of the lactose operon (lacO) [34] were examined by fluorescence microscopy. As shown in Figure 2D, GFP-LacI segregated to both spindle pole bodies (marked by the spindle pole marker, Spc29-CFP) in a similar manner to wild type cells. We also examined for chromosome mis-segregation in *glc7-E101Q *cells following several growth cycles using a sectoring assay [35]. Wild type, *glc7-E101Q *and *ame1-6 *(positive control) strains lacking a functional *ADE2 *gene (*ade2Δ-101*) were transformed with a centromeric fragment of chromosome III containing the *SUP11 *gene (Circle III/*SUP11*) and analyzed for loss of the fragment after 4-day incubation on YPD medium lacking adenine. The *ame1-6 *strain exhibited frequent chromosome loss as indicated by the appearance of red sectors in the colonies (Figure 2E). In contrast, *glc7-E101Q *and wild type colonies induced negligible loss of the Circle III/*SUP11 *fragment, and the vast majority of colonies remained white in coloration (Figure 2E). We also tested *glc7-E101Q *cells for sensitivity to the microtubule-disrupting agent, benomyl. When compared to the wild type, *glc7-E101Q *cells had consistently slower growth on both DMSO control and benomyl plates. However, the slow growth of *glc7-E101Q *was not exacerbated by benomyl treatment (Figure 2F).

To investigate the possibility that the slow growth phenotype of *glc7-E101Q *cells might be the result of either protein instability or changes in protein modifications to Glc7, whole cell extracts were examined by Western blot analysis. Protein extracts from strains expressing Glc7-ProA or *glc7-E101Q*-ProA fusion proteins were prepared from asynchronous cells (1D immunoblot) or from G1, S and G2/M-arrested cells (2D immunoblot) and examined using anti-ProA antibodies. The abundance of the *glc7-E101Q*-ProA mutant was not reduced relative to wild type Glc7-ProA by 1D immunoblot analysis (Additional File 2A). Furthermore, in each case, a single isoform was identified for Glc7-ProA and *glc7-E101Q*-ProA in extracts derived from G1, S and G2/M arrested cells (Additional File 2B). These findings indicate that the slow growth of *glc7-E101Q *strains was not the result of protein instability or the accumulation of aberrant modifications to the Glc7 protein.

### *glc7-E101Q *mutant is impaired in glucose metabolism

**Figure 3:**
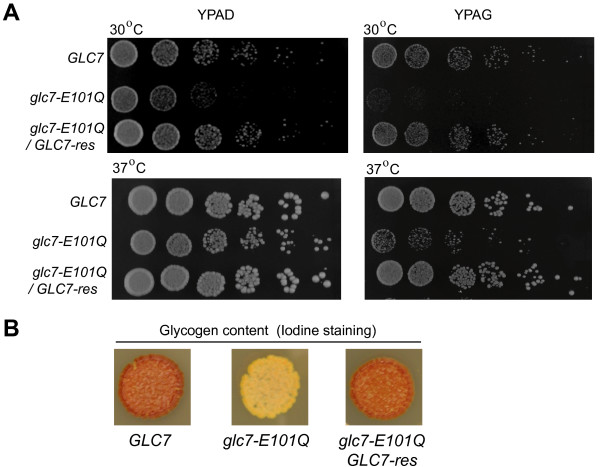
***glc7-E101Q *mutant is impaired in glucose metabolism**. (A) Growth of wild type (*GLC7*), mutant (*glc7-E101Q*) and rescue (*glc7-E101Q/GLC7-res*) strains was examined by spotting assays (5-fold serial dilutions of 1.0 × 10^6 ^cells/ml; 5 μl/spot) grown in YPAD or YPAG for 48 hr at 30°C and 37°C. (B) Glycogen content was assessed in wild type (*GLC7*), mutant (*glc7-E101Q*) and rescue (*GLC7-res*) strains by iodine staining. Brown color correlates with glycogen content.

Glc7 has a well-established role in glucose metabolism. The first conditional allele of *glc7 (glc7-1) *was isolated as a consequence of its defect in glycogen synthesis in the presence of glucose [36], a phenotype that has, similarly, been observed for other *glc7 *mutants [22,36]. The failure to accumulate glycogen has been linked to impaired de-phosphorylation of the glycogen synthase, Gsy2, by Glc7 and its targeting subunit, Gac1 [15,37]. Glc7 also plays an important role in glucose repression, a process that (in the presence of glucose) leads to transcriptional repression of genes involved in the utilization of alternative carbon sources such as sucrose, galactose and maltose. Conditional *glc7 *mutants such as *glc7-T152K *[25,38], *glc7-F256A, glc7-F292A, glc7-E241 L242 *[16] exhibit defects in glucose repression that is due, in part, to an impaired ability of the Glc7 mutant to associate with the targeting subunit Reg1. In the presence of glucose, Glc7-Reg1 has been shown to de-phosphorylate and thereby, inhibit Snf1 kinase, which is responsible for the transcription of several glucose-repressed genes [25,39]. We observed that *glc7-E101Q *cells had a more significant growth defect in limited glucose (2% glycerol, 0.08% glucose; YPAG) relative to its growth in rich medium (2% glucose; YPAD). Growth of *glc7-E101Q *cells on both YPAD and YPAG was similar at 30°C and 37°C, indicating the *glc7-E101Q *mutation does not induce temperature-sensitivity (Figure 3A). Glycogen content was assessed by iodine staining [40] and revealed impaired glycogen accumulation for *glc7-E101Q *cells (Figure 3B). Both the impaired growth and the glycogen accumulation defects of *glc7-E101Q *were restored to wild type levels in strains that expressed an un-linked copy of wild type *GLC7:URA3 *inserted at the *ura3 *locus (*GLC7-res*).

To rule out the possibility that the poor growth of *glc7-E101Q *observed on YPAG is the consequence of a petite phenotype resulting from mitochondrial mis-function, tetrad analysis was performed on diploid cells heterozygous for *glc7-E101Q *in a genetic background homozygous for a defective *ade2Δ-101 *gene. In the absence of adenine, *ade2Δ-101 *cells accumulate P-ribosylaminoimidazole, an intermediate of the adenine synthesis pathway. Mitochondrial oxidation of this intermediate results in a red pigment which colors colonies [41]. We observed *glc7-E101Q *spore colonies consistently accumulated this red pigment on medium lacking adenine demonstrating the presence of functional mitochondria (data not shown). Taken together, these results support that the *glc7-E101Q *mutation is a stable recessive allele and exhibits impaired catalytic activity *in vivo*.

### SGA analysis of *glc7-E101Q *reveals a broad genetic network encompassing Glc7-regulated processes

**Figure 4:**
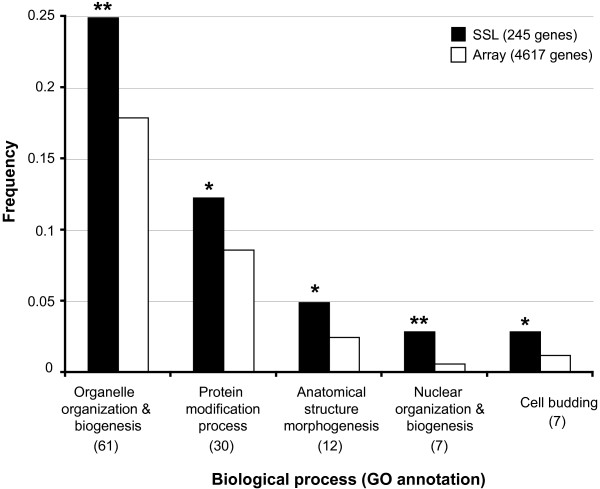
**Biological processes overrepresented in *glc7-E101Q *SSL genes**. Indicated are the frequencies of high-level GO terms represented in the set of SSL genes (closed bars) that were significantly enriched (*p *< 0.01, **; *p *< 0.05, *) when compared to those represented in the set of array genes (open bars). The number of genes identified in each GO process is shown in parentheses.

In order to expand the genetic network of Glc7 and to identify new candidate genes involved in Glc7-dependent processes, we screened ~4,600 non-essential genes for synthetic interactions with *glc7-E101Q *using the SGA methodology (Additional Files 3 and 4). Two-independent genome-wide screens revealed a total of 786 candidate synthetic sick/lethal (SSL) interactions (Additional File 4). To confirm SSL genes identified, we utilized a modified spot assay version of random spore analysis described previously [13]. Since Glc7 has been shown to regulate sporulation [19,22,42] and meiosis [43,44] we wished to rule out the possibility that genes identified as SSL with *glc7-E101Q *may have resulted from meiotic and/or sporulation defects due to haploinsufficiency for *GLC7*. To circumvent this problem, random spore analysis was performed using a query strain of *glc7-E101Q *that contained *GLC7:URA3 *under control of the endogenous *GLC7 *promoter inserted in the *ura3 *locus (*GLC7-res*) (see Methods and Additional File 3). Using this methodology, mating, sporulation and selection of *MATa *haploid progeny were performed under conditions in which wild type *GLC7 *was co-expressed in the *glc7-E101Q *mutant. Following the selection of *MATa *progeny, cells expressing wild type *GLC7 *were eliminated by plating on 5-fluoro-orotic acid (5-FOA) medium which prevents the growth of cells expressing a functional *URA3 *gene [45]. Plate scoring from the random spore analysis confirmed 245 genes as SSL with *glc7-E101Q*. The SSL genes were then grouped into functional classes according to their biological process GO (Gene Ontology) annotations deposited in the *Saccharomyces *Genome Database (GO-slim mapping table, downloaded on June 4^th^, 2008). Additional File 5 lists the 494 annotations encompassing 35 high-level GO terms for the 245 SSL genes. Using a hypergeometric distribution model [46], the frequency distribution of the GO terms identified for SSL genes was compared to those represented in the deletion array and examined for enrichment of particular biological processes (Figure 4 and Additional File 6). The set of SSL gene annotations was significantly enriched for 5 GO-terms: Organelle Organization and Biogenesis (*p *< 0.01); Protein modification process (*p *< 0.05); Anatomical Structure Morphogenesis (*p *< 0.05); Nuclear Organization (*p *< 0.01); and Biogenesis and Cell Budding (*p *< 0.05). Since Glc7 has been shown to play a role in all these processes, this suggests that the *glc7-E101Q *mutant is defective in multiple aspects of the Glc7 function.

To reveal the processes buffered by *GLC7*, 163 of 245 genes interacting with the *glc7-E101Q *mutant were manually grouped into 12 functional categories (Table 2). The majority of these genes are implicated in critical cellular processes such as chromosome segregation, nutrient-sensing, stress response and mitochondrial function. The information currently available on the remaining set of genes is too fragmental to allow classification in any of the listed categories (Additional File 5).

**Table 2:** Functional processes of 163 genes showing SSL interactions with *glc7-E101Q*

Functional category	Genes
Cell polarity	*AXL2, BUD4*

Chromosome segregation	*BAG7, BIM1, BNR1, BOI2, BUB1, BUB3, BUD14, CIK1, CTF8, KAP120, KAR3, MAD1, MAD2, MCM22, NUP53, NUP84, NUP133, REC8, ROM2, SXM1, TUB3*

DNA damage	*EXO1, IES2, LIF1, LSM12, MRC1, PMS1, RAD4, RAD14, RFX1, RSC2, TDP1, UNG1, YFR017C, YPR147C*

Histone modification	*ASF1, CHD1, DOT1, FPR4, PHO23, RTT109, UAF30*

Lipid metabolism	*ANT1, BUD25, ERG5, OPI3, OSH3, PCS60, PEX6, PEX29, PEX31, PLB1, PSD1, SCS3*

Mitochondrial function	*ATP4, ATP12, CBR1, COX19, CYC7, FMP42, HAP2, IMP1, MDM34, MIR1, MRS4, MSK1, MTF1, PDX1, PET122, PUT2, RIM1, RML2, RSM18, SCO1, VPS73, YPT11, YBL095W, YGL059W, YGR021W, YGR287C, YHR162W, YJR039W, YNL320W*

Nutrient sensing and stress signaling	*ATG5, ATG15, BAP2, CKB1, CKB2, GPA2, GPH1, HIT1, HXT17, MCA1, MCH4, NST1, NVJ1, ORM2, SAK1, SFL1, SIP5, SPG5, SSK1, TDH3, TPK1, UGA1, URM1, VAC8, WHI2, YBR235W, YJL193W, YNR029C*

Phosphatase genes	*PPM1, PPZ1, RTS3*

Protein modification and degradation	*KTR1, KTR3, KTR4, NAT3, NPL4, OTU1, PMT5, SLX8, UMP1, USA1*

Protein synthesis	*RPL6B, RPL12A, RPL19A, RPL24A, RPL37A, RPS1A, RPS16A, RPS19A, RPS25A, YBL054W*

SAM^(1) ^synthesis	*BUD23, NCL1, RMT2, SPE2, TRM1*

Transcription	*CAF120, CDC40, DAT1, DST1, ELP2, HMS2, KTI12, LSM6, NUT1, RTT103, SSN2, SSN8, SUM1*

Vesicular transport	*ARF1, BTS1, COG5, END3, ENT5, GSG1, GYP8, MST27, UBP3*

^(1)^: S-adenosylmethionine

#### Chromatin remodeling, chromosome segregation, DNA damage, cell polarity and cytokinesis genes

A set of genes encoding histone-modifying proteins with a role in chromatin remodeling and transcription show SSL interaction with *glc7-E101Q*. This group includes the histone H3 acetyltransferase *RTT109/KAT11*, the histone H3 methylase *DOT1/KMT4*, the component of the Rpd3 histone deactylase *PHO23*, the subunit of the SAGA/SLIK complexes *CHD1 *involved in histone acetylation, the peptidyl-prolyl cis-trans isomerase *FPR4 *involved in histone H3 and H4 folding, the nucleosome remodeling factor *ASF1*, and the histone-associated factor *UAF30*. These results are in agreement with a previous observation that *doc1*Δ *glc7-127 *double mutants exhibited a slow growth phenotype due to mis-regulation of histone H3 phosphorylation [47]. In addition, the loss of *SET1*, which encodes a histone methyltransferase, was also shown to cause lethality in *glc7-127 *cells [48]. Collectively, these findings indicate that correct histone modification is required for the survival of *glc7 *mutants.

Genes with a role in DNA damage included components of chromatin remodeling complexes (*IES2 *and *RSC2*), the checkpoint signaling gene, *MRC1*, subunits of DNA-repair complexes (*EXO1*, *LIF1*, *PMS1*, *RAD4*,*RAD14*, *TDP1 *and *UNG1*) and DNA-damage responsive genes (*LSM12*, *RFX1*, *YFR017C *and *YPR147C*). This set of genes emphasizes the requirement for proper chromosome segregation in *glc7-E101Q *cells and the critical role of the DNA-repair machinery during mitosis. Several other genes identified indicate that Glc7 plays a critical role in chromosome segregation and cytokinesis. Genes in these categories include regulators of microtubule dynamics (the microtubule +TIP protein *BIM1*, a-tubulin *TUB3*, and the Glc7 regulatory subunit *BUD14*) and the cytokinetic actomyosin ring (the formin *BNR1*, anillin-like protein *BOI1*, and two regulators of Rho1 GTPase *ROM2 *and *BAG7 *[49,50]). In addition, *AXL2 *and *BUD4 *were identified which share a physical interaction and are implicated in cell polarity and cell-cycle control [51]. Other genes involved in chromosome segregation included chromatid cohesion genes (*CTF8 *and *REC8*), the mini-chromosome maintenance gene, *MCM22*, cytokinesis genes (*CIK1 *and *KAR3*), components of the spindle checkpoint (*BUB1*, *BUB3*, *MAD1 *and *MAD2*), subunits of the nuclear pore complex (*NUP53*, *NUP84 *and *NUP133*) and karyopherin genes (*KAP120 *and *SXM1*). The identification of a wide-spanning range of genes involved in chromosome segregation supports that Glc7 plays a prominent role in buffering against chromosome mis-segregation. This is supported by previous findings that loss of *BUD14 *[52] or mutation of the kinetochore protein, *DAM1*, [53] caused lethality of conditional *glc7 *mutants. Furthermore, other data supports that Glc7 plays a key role in regulating the attachment of kinetochore microtubules to chromosomes. It is postulated that this regulation occurs via Glc7-dependent de-phosphorylation of kinetochore-localized proteins, such as Ndc10 and other yet to be identified substrates [29,30,32].

#### Nutrient-sensing, stress response and mitochondrial genes

In accordance with Glc7's prominent metabolic role, particularly glycogen accumulation and glucose repression, several genes involved in nutrient sensing, metabolism and stress response were identified. *SIP5 *and the Snf1-activating kinase *SAK1*, which are both involved in the regulation of Snf1 kinase, were required for growth of *glc7-E101Q*. Sip5 physically interacts with both Reg1-Glc7 and Snf1, and is thought to regulate the interaction between Glc7 and Snf1 [39]. Other nutrient sensing genes included *GPH1 *(glycogen phosphorylase), *TDH3 *(glyceraldehyde-3-phosphate dehydrogenase), *GPA2 *(a-subunit of the glucose sensor G-protein), *TPK1 *(cAMP-dependent kinase involved in the Ras-cAMP signaling pathway), *SSK1 *(HOG pathway kinase), *CKB1 *and *CKB2 *(β-and β'-casein kinase subunits) and various transport proteins (*BAP2*, *HXT17*, *MCH4*, *YBR235W *and *YJL193W*). Genes required for growth under stress conditions (*ATG5*, *ATG15*, *HIT1*, *MCA1*, *NST1*, *NVJ1*, *ORM2*, *SFL1*, *SPG5*, *UGA1*, *URM1*, *VAC8*, *WHI2 *and *YNR029C*) are also in this group. These findings indicate that nutrient sensing and stress signaling pathways are required for the growth of the *glc7-E101Q *mutant. This group of interactions can be related to a previous observation that mutation of *RHR2*, encoding the DL-glycerol-3-phosphatase, is lethal when introduced in a *glc7-Y170 *background [54]. In addition, epistasis and transcriptional analyses have shown a role for Bud14-Glc7 in the regulation of Msn2, a transcription factor controlling the expression of **st**ress-**r**esponse **e**lement (STRE)-genes [55].

Genes with a role in mitochondrial activity and inheritance included subunits of the ATP synthase (*ATP4 *and *ATP12*), genes required for cytochrome-c oxidase function (*COX19*, *CYC7 *and *SCO1*), genes involved in mitochondrial replication (*RIM1*)-transcription (*HAP2*, *MTF1 *and *PET122*) and -translation (*MSK1*, *RML2 *and *RSM18*), genes required for mitochondrial homeostasis/metabolism (*CBR1*, *MIR1*, *MRS4 *and *PDX1*) and genes with a role in mitochondrial morphology and distribution (*IMP1*, *MDM34 *and *YPT11*). This group also contains genes that localize to the mitochondria (*FMP42*, *PUT2*, *VPS73*, *YBL095W*, *YGL059W*, *YGR021W*, *YGR287C*, *YHR161W*, *YJR039W *and *YNL320W*) and may be required for optimal mitochondrial function. Thus, mitochondrial function is essential when PP1 function is compromised.

#### Transcription, protein synthesis and protein regulation genes

Genes involved in transcription included transcription factors (*CAF120*, *DAT1*,*DST1*, *HMS2*, and *SUM1*), elongator complex components (*ELP2 *and *KTI12*), mediator complex components (*NUT1*, *SSN2 *and *SSN8*), a transcription termination factor (*RTT103*) and RNA processing factors (*CDC40 *and *LSM6*). Protein synthesis genes included components of the large ribosomal subunit (*RPL6B*, *RPL12A*, *RPL19A*, *RPL24A *and *RPL37*), components of the small ribosomal subunit (*RPS1A*, *RPS16A*, *RPS19A *and *RPS25A*) and *YBL054W*, which is putatively involved in rRNA synthesis. Other genes were identified with various roles in protein modification such as protein glycosylation (*KTR1*, *KTR3*, *KTR4 *and *PMT5*), ubiquitin-regulated protein degradation (*NPL4*, *OTU1*, *SLX8*, *UMP1 *and *USA1*) and protein phosphorylation (*PPZ1*, *PPM1 *and *RTS3*). All these genes likely play a role in the expression of and/or the modification/turnover gene products essential for processes buffered by Glc7.

The synthetic lethal interaction of *glc7-E101Q *with the *PPZ1 *phosphatase and two PP2A subunits (*PPM1 *and *RTS3*) suggests an interplay between these phosphatases. Few synthetic lethal interactions are currently available for *PPZ1*, *PPM1 *and *RTS3*. However, it was interesting to note that *glc7-E101Q *and *PPZ1 *share synthetic lethal interactions with both *BIM1 *and *BUB3*. In addition, it has been previously suggested that an exchange of regulatory subunits between Glc7 and PPZ phosphatase may be the basis for the observed synthetic lethal interaction of *glc8 *with *glc7 *mutants and *ppz1 ppz2 *[56]. These observations support that signaling cross-talk occurs between these phosphatases.

#### Vesicle transport, lipid metabolism and S-adenosylmethionine synthesis genes

Genes with a role in vesicle transport included *ARF1 *(coat formation small GTPase), *BTS1 *(geranygeranyl diphosphate synthase required for the interaction of small GTPases with vesicle/organelle membranes), regulators of Golgi vesicle targeting and fusion (COG complex subunit *COG5*, the TRAPP subunit *GSG1*, and the Ypt1 GTPase Activating Protein, *GYP8*) and regulatory proteins involved in endocytosis (actin cytoskeleton regulator, *END3*, phosphoinositide-binding protein, *ENT5*, and the coat regulators, *MST27 *and *UBP3*). These interactions may indicate a requirement for the transport of specific cargo(s) in the *glc7-E101Q *mutant that are required for viability. Alternatively, they may reflect a more direct role for Glc7 in the regulation of vesicle transport. This is supported by previous studies that indicate a regulatory function for Glc7 on components of vesicle trafficking. For example, Glc7 and its targeting subunit, Scd5, were shown to regulate Pan1, an actin regulatory protein involved in endocytosis. Interestingly, both Pan1 and End3 (identified in our SGA analysis) exhibited physical interactions with Scd5, implicating them in Glc7 recruitment to sites of endocytosis [57]. In addition, a separate study demonstrated a role for Glc7 in the regulation of SNARE protein complexes that are required for vesicle-membrane fusion [58].

Lipid metabolism genes identified included those functioning in ergosterol metabolism (*ERG5 *and *OSH3*), fatty acid metabolism (*ANT1 *and *PCS60*), phospholipid metabolism (*OPI3*, *PLB1*, *PSD1 *and *SCS3*) and a set of genes for peroxisomal proteins (*PEX6*, *PEX29 *and *PEX31*). The intimate synthetic lethal relationships between lipid metabolism, mitochondrial function and vesicle transport genes is probably the basis for this set of SSL interactions.

Genes involved in S-adenosylmethionine (SAM) -dependent processes included methyltransferases (*BUD23*, *NCL1*, *RMT2 *and *TRM1*) and the SAM decarboxylase *SPE2*. The deletion of these genes likely impacts on SAM pool and this, in turn, affects Glc7-dependent processes that utilize this co-factor such as histone methylation, ergosterol synthesis and phospholipid synthesis.

### Network analysis of the Glc7 interacting genes

**Figure 5:**
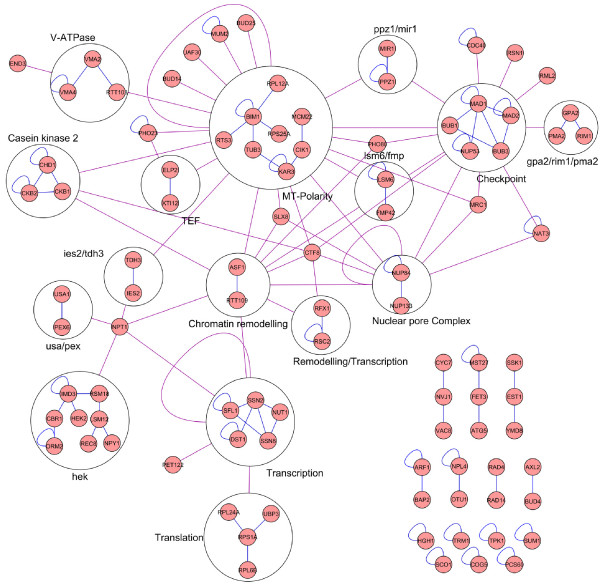
**A network of protein-protein and genetic interactions among *glc7-E101Q *SSL genes**. Genes showing SSL interaction with *glc7-E101Q *(nodes) were grouped in modules based on their physical interaction profiles (blue edges). The genetic interactions interconnecting modules or connecting modules and single nodes are depicted as red edges.

We performed a network analysis of the *glc7-E101Q *SSL genes by examining protein-protein and genetic interactions available from the BioGRID database (version 2.0.41, released on June 1, 2008 [26]). This analysis revealed 95 genes that are interconnected through 99 and 212 protein-protein and genetic interactions, respectively. Based on protein-protein interactions, we identified 23 distinct molecular modules involving 72 *GLC7 *genetic interactors (Figure 5). These modules are components of molecular machines involved in microtubule-based polarity, the spindle checkpoint, chromatin remodeling and transcription, translation, regulation of casein kinase 2, regulation of the V-ATPase, nuclear pore complex and nucleo-vacuolar transport. Some modules are interconnected by genetic interactions among their components (Figure 5, red edges). For example, components of the "microtubule-based polarity", the "spindle checkpoint" and the "nuclear pore complex" modules show genetic interactions. This reflects how these modules cooperate during mitosis. In some cases, two modules are connected by genetic interaction with a gene that is not part of any module. For example, *CTF8 *and *SLX8*, both playing a role in the control of DNA replication, connect via genetic interactions to the "microtubule-base polarity", "nuclear pore complex" and "remodeling/transcription" modules. Finally, some modules, such as "*NPL4*-*OTU1*" (whose products are involved in polyubquitin-mediated protein degradation) and "*AXL2*-*BUD4*", are not connected to any other module. This probably reflects that the map for genetic interactions is still incomplete and that some genes showing SSL interaction with *glc7-E101Q *have not yet been screened by the SGA. Additional SGA screens will be required to obtain a comprehensive interaction profile for these genes.

## Discussion

A number of studies support that Glc7 plays a critical role in the regulation of several cellular processes such as cell polarity, chromosome segregation, cytokinesis, and cell cycle control [1-4]. However, the precise role of Glc7 in the majority of these processes and the identity of Glc7 substrates involved in the regulation of these events remains poorly understood. The lack of information regarding Glc7's signaling network is due, in part, to a large disconnect between genetic and proteomic data available. Currently, the number of protein-protein interactions reported greatly exceeds genetic interactions [26,59,60]. The genetic interaction space, however, is much more dense than the physical interaction network, and the number of SSL combinations for a given gene is estimated to be 4-times that of protein-protein interactions [13]. The high number of physical interactions and diverse phenotypes of conditional *glc7 *mutants suggest Glc7 has a large and complex SSL interaction network.

In order to expand the genetic interaction data set for Glc7, we examined a novel catalytic mutant, *glc7-E101Q*, for synthetic lethal interactions using SGA. Existing conditional *glc7 *alleles contain mutations outside of the Glc7 catalytic domain that can alter the binding of regulatory subunits [1,18,19,22,25], potentially resulting in allele-specific genetic interaction patterns. To achieve our goal to globally map the *GLC7 *genetic interactions, a hypomorphic *glc7 *allele was constructed. Based on 3-dimensional modeling and previous structure-function studies, the SGA query mutant *glc7-E101Q *was predicted to exhibit reduced catalytic efficiency while maintaining structural similarity to wild type *GLC7*. That two iron transporter genes (*FET3 *and *MRS4*), were found in our SGA analysis suggested that a normal iron supply is important for the normal growth of the *glc7-E101Q *mutant, and is consistent with the idea that this allele is catalytically compromised due to altered metal cofactor binding. Furthermore, phenotypic analyses indicated *glc7-E101Q *is a stable recessive hypomorphic allele. Similar to some conditional *glc7 *mutants, *glc7-E101Q *cells exhibited impaired growth in glucose-limited medium and defects in glycogen accumulation [16,22,36]. However, in contrast to other *glc7 *mutants that have been shown to have chromosome mis-segregation and cell cycle progression defects (e.g.: *glc7-10 *[1,32], *glc7-129 *[29] and *glc7-Y170 *[31]), *glc7-E101Q *cells had no appreciable cell cycle delay or chromosome loss. These observations suggest a threshold effect may occur in *glc7-E101Q *cells, such that weakened catalytic efficiency of Glc7 is tolerated and/or compensated at differing levels depending on the cellular process and the demand for Glc7 activity.

SGA analysis of *glc7-E101Q *resulted in a large number (786) of SSL hits in two separate screens. We expect that the large number of genes identified be due, at least in part, to the haploinsufficiency observed for *glc7-E101Q *mutants, which resulted in reduced spore viability and placed constraints on colony scoring. To solve this problem and generate a high confidence dataset, we modified the confirmation procedure by creating a second query strain that expressed a selectable copy of wild type *GLC7 *(*GLC7-res*). Sporulation, meiosis and growth of haploid progeny were performed in *glc7-E101Q *cells expressing wild type *GLC7 *inserted at the *URA3 *locus. Pinning on counter-selective medium then eliminated haploid cells expressing the wild type *GLC7 *allele. Using this strategy, false positive hits were rejected and 245 hits were confirmed as true SSL interactions. We anticipate this strategy may be applicable to confirm SGA screens of other query genes that interfere with sporulation or spore germination.

Genes showing synthetic sick/lethal interactions with *glc7-E101Q *were grouped in 12 functional classes. Although only one (*BUD14*) of 7 previously reported SSL interactions (*BUD14*, *DAM1*, *DOC1*, *GLC8*, *RHR2*, *SET1 *and *SLT2*) for *GLC7 *was identified in our analysis, a number of established Glc7-mediated processes were captured in our screen. In particular, sets of genes fall into functional classes encompassing the 7 previously reported *GLC7 *SSL interactions, which are chromatin remodeling, chromosome segregation/cytokinesis, and nutrient sensing/metabolism. In addition, certain SSL genes identified highlight events that appear to be directly regulated by Glc7. These include genes involved in glucose repression (*SIP5*) [39], chromosome segregation (*BUD14*) [18] and endocytosis (*END3*) [57]. Lastly, our SGA analysis of *glc7-E101Q *provides a rich data set of genetic interactions that expands on previously known Glc7-regulated processes, and the *glc7-E101Q *synthetic interaction pattern reveals new facets of Glc7 function. For example, the requirement for mitochondrial and phospholipid metabolism genes when Glc7 function is compromised suggests a role for Glc7 in these processes.

## Conclusion

To uncover the genetic interaction network of the essential PP1 gene *GLC7*, a novel catalytic mutant allele, *glc7-E101Q*, was used in an unbiased genome-wide screen for SSL interactions. We found 245 genes whose deletion was detrimental for the growth of *glc7-E101Q*. Functional grouping of these genes and analysis of their physical and genetic interaction expand the genetic network of established Glc7-regulated processes and suggest novel regulatory roles for Glc7.

## Methods

### Media, growth conditions and strain manipulations

Yeast strains (Table 1) were created through PCR-based transformation [61,62]. Media (rich media: YPAD and YPD (2% glucose), YPAG (3% glycerol with 0.08% glucose); synthetic media: SC and SD) were prepared as previously described [63]. Standard methods for culture of yeast strains and integrative transformation, mating, sporulation and tetrad analysis was performed as previously described [63]. SGA analysis was performed in strains derived from the S288C diploid BY4743 [64]. All other analyses were performed in strains derived from the S288C diploid Y270 [65].

**Table 1:** Yeast strains used in this study

**Strain**	**Genotype**	**Source**
BY4743	*MATa/α ura3Δ0/ura3Δ0 leu2Δ0/leu2Δ0 his3Δ1/his3Δ1 * *LYS2/lys2Δ0 met15Δ0/MET15*	[64]

Y270	*MATa/MATα ura3-52/ura3-52 lys2-801/lys2-801 ade2-101/* *ade2-101 trp1Δ1/trp1Δ his3Δ200/his3Δ200*	[65]

Y5563	*MATa can1Δ::MFA1pr-HIS3 lyp1Δ his3Δ1 leu2Δ0 ura3Δ0 met15Δ0*	C Boone

ΔArrayORF	*MATa orfΔ::KanMX4 LYS2 his3Δ1 leu2Δ0 met15Δ0 ura3Δ0*	[71]

YNS2	*MATα ura3-52 lys2-801 ade2-101 trp1Δ1 his3Δ200*	This study

YNS3	*MATa ura3-52 lys2-801 ade2-101 trp1Δ1 his3Δ200*	This study

YNS19	*MATa/MATα glc7-D91N:KanMX4/GLC7 ura3-52/ura3-52 * *lys2-801/lys2-801 ade2-101/ade2-101 trp1Δ1/ trp1Δ1 * *his3Δ200/his3Δ200*	This study

YNS23	*MATa GLC7:ProA:KanMX4 ura3-52 lys2-801 ade2-101 * *trp1Δ1 his3Δ200*	This study

YNS26	*MATα glc7-E101Q:ProA:KanMX4 ura3-52 lys2-801 ade2-101 * *trp1Δ1 his3Δ200*	This study

YNS27	*MATa glc7-E101Q:ProA:KanMX4 ura3-52 lys2-801 ade2-101 * *trp1Δ1 his3Δ200*	This study

YNS44	*MATα GLC7:ProA:KanMX4 ura3-52 * *lys2-801 ade2-101*	This study

YNS53	*MATa/MATα glc7-E101Q:KanMX4/GLC7 ura3-52/ura3-52 * *lys2-801/lys2-801 ade2-101/ade2-101 trp1Δ1//trp1Δ1 * *his3Δ200/his3Δ200*	This study

YNS90	*MATα glc7-E101Q:kanMX4 ura3-52 lys2-801 ade2-101 trp1Δ1 * *his3Δ200*	This study

YNS91	*MATa glc7-E101Q:KanMX4 ura3-52 lys2-801 ade2-101 * *trp1Δ1 his3Δ200*	This study

YNS98	*MATα glc7-E101Q:NatMX4 can1Δ::MFA1pr-HIS3 lyp1Δ * *his3Δ1 leu2Δ0 ura3Δ0 met15Δ0*	This study

YNS99	*MATa ura3-52::GLC7:URA3 lys2-801 ade2-101 trp1Δ1 his3Δ200*	This study

YNS100	*MATα glc7-E101Q:NatMX4 ura3-52::GLC7:URA3 * *can1Δ::MFA1pr-HIS3 lyp1Δ his3Δ1 leu2Δ0 ura3Δ0 met15Δ0*	This study

YNS101	*MATa his3-11,15::GFP:LacI:HIS3 lacO:URA3, * *SPC29:CFP:kanMX4 lys2-801 ade2-101 trp1Δ1*	This study

YNS102	*MATa glc7-E101Q:kanMX4 his3-11,15::GFP:LacI:HIS3 * *lacO:URA SPC29:CFP:kanMX4 lys2-801 ade2-101 trp1Δ1*	This study

YNS128	*MATa/MATα glc7-E101Q:KanMX4/GLC7 ura3-52/ ura3-52:* *:GLC7:URA3 lys2-801/lys2-801 ade-101/ade2-101 trp1Δ1/trp1Δ1 * *his3Δ200/his3Δ200*	This study

YNS138	*MATa glc7-E101Q:KanMX4 ura3-52::GLC7::URA3 lys2-801 ade2-101 * *trp1Δ1 his3Δ200*	This study

YNS141	*MATα glc7-E101Q:KanMX4 ura3-52::GLC7::URA3 lys2-801 ade2-101 * *trp1Δ1 his3Δ200*	This study

### Mutagenesis of Glc7 and strain construction

A 1.2 kb PCR product containing 3' sequence of the *GLC7 *ORF was cloned into pBSK using *KpnI *and *NotI *to create pGLC7-1. The *KANMX4 *selection cassette was amplified by PCR from pFA6A-KANMX4, digested with *NotI *and *SacI *and cloned into pGLC7-1, creating pGLC7-KANMX4. This construct was used as a template for PCR-based mutagenesis of the catalytic domain at codon 273 (D91N: GAT to AAT) and codon 303 (E101Q: GAG to CAG). A silent restriction site (*AvrII *in D91N and *HindIII *in E101Q) was introduced into the mutagenic 5' primer to mark the mutation. Mutagenic PCR products were transformed into Y270 as previously described [65], and G418-resistant transformants selected for on YPAD containing 200 μg/ml G418 (GIBCO). Integration into the *GLC7 *locus was confirmed by amplification of a PCR product that spanned the ORF and the *KANMX4 *cassette and by restriction digest with *AvrII *or *HindIII*. Finally, the presence of a single point mutation (D91N or E101Q) in the ORF and no others was confirmed by sequencing. Diploid strains heterozygous for D91N (YNS19) and E101Q (YNS53) mutations were sporulated, and haploid *glc7-E101Q:KANMX4 *MATa and MATa segregants (YNS91, YNS90) isolated for phenotypic analyses. A similar cloning strategy was used to create ProA fusions of both wild type *GLC7 *and *glc7-E101Q*. A Glc7 PCR product lacking the endogenous stop codon was cloned into pBSK (pGLC7-2) and the ProA and *KANMX4 *selectable markers from pFA6A-ProA-KANMX4 [66] integrated using *NotI *and *SacI*, creating pGLC7-ProA. This construct was used for PCR directed mutagenesis and transformation into Y270 as described above, sporulated and haploid segregants (YNS26, YNS27) isolated for the analysis of protein stability. Wild type GLC7-proA strains were derived in a similar manner (YNS23, YNS44).

For *glc7-E101Q *rescue experiments, a *GLC7 *was cloned into the integrative plasmid pRS306 by first amplifying a PCR product from genomic DNA that contained the entire *GLC7 *ORF and 0.5 kb 5' promoter sequence and 0.3 kb 3' UTR, digested with *KpnI *and *SacI *and ligated into pRS306 [67], resulting in pGLC7-3. pGLC7-3 was linearized with *StuI*, and transformed into the *ura3 *locus of the heterozygous diploid strain YNS53. Integration at the *ura3 *locus resulted in wild type copy of *GLC7 *marked with *URA3 *(*GLC7-res*; strains YNS138, YNS141) on the opposite arm (116167–116970) of chromosome V relative to the *GLC7 *locus (432491–433954). In ~40 meiotic events *GLC7-res *segregated as an un-linked gene (28TT : 3NPD : 9PD) independently of *GLC7 *and *glc7-E101Q*.

To create the SGA query strain, YNS98, a *NATMX4 *PCR product with homology to the *GLC7 *3' UTR sequences was amplified by PCR from p4339 [12], and used to replace the *KANMX4 *cassette in YNS90. Genomic DNA of the resulting strain was then used to amplify a PCR product containing the E101Q mutation and the *NATMX4 *cassette. This PCR product was transformed into Y5563 according to previously described methods [13]. To create the query strain for random spore analysis (YNS100), a *MATa glc7-E101Q*:*NATMX4*/*GLC7-res *strain was first isolated by tetrad dissection from a heterozygous diploid (YNS99 × YNS98) and then backcrossed to Y5663.

### Growth assays, FACS and glycogen staining

To assess cell growth, wild type (YNS3) and *glc7-E101Q *(YNS91) and GLC7-*res *(YNS138) strains were grown to mid-log phase in YPAD, diluted to 5.5 × 10^5 ^cells/ml (~0.05 OD units) and incubated at 30°C for a total of 7 hours. Cells were collected at 1-hour intervals, and cell density determined using a hemocytometer. For spotting assays, log phase cells grown in liquid YPAD were diluted to 1.0 × 10^6 ^cells/ml (~0.1 OD units) and 5-fold serial dilutions spotted (5 μl/spot) to YPAD or YPAG plates and incubated for 2–3 days at 18°C, 25°C, 30°C and 37°C. To determine benomyl-sensitivity, cells were examined in parallel with benomyl-sensitive (*tub1-1*) and resistant (*tub2-104*) strains [68,69] on 10 μg/ml benomyl or DMSO plates incubated for 2–3 days at 30°C. Cell cycle progression of wild type and *glc7-E101Q *strains was determined by FACS analysis as previously described [70]. Fixed cells from the FACS analysis were also used for bud index scoring, and the proportion of un-budded, small-budded (bud <50% mother cell) and large-budded cells determined. Cells were pre-treated with Zymolase (20 μg/ml) to complete cell separation. Glycogen staining was performed as previously described [40]. Briefly, after 2-day incubation at 30°C, plates were exposed to iodine crystals (Sigma) for 1 minute, removed for 15 sec and exposed again for 2 min. Digital images were captured immediately after exposure to iodine.

### Analysis of chromosome segregation

#### Microscopy

*MAT*a wild type (YNS101) and *glc7-E101Q *mutant (YNS102) strains that expressed Spc29-CFP (SPB marker), GFP-LacI and centromeric (CEN15) lacO repeats [34] were isolated from heterozygous diploids. Cells were incubated in 5 μg/ml a-factor for 3 hrs at 30°C, washed and released in YPAD and collected every 10 min (80 min total). Samples were fixed for 5 min in 4% formaldehyde, washed three times in PBS and imaged using a Nikon TE200 inverted microscope equipped with a 100 × 1.4 NA objective mounted on PE piezo z-drive/Improvision Orbit controller and Hammamatsu ORCA-ERG camera. Image stacks (0.5 μm sections, 10–12/image) were acquired using Volocity 4.0 and image deconvolution with Volocity 4.0 Restoration.

#### Sectoring assay

*MAT*a wild type (YNS3), *glc7-E101Q *(YNS91) and *ame1-6 *(positive control for chromosome loss; Vogel unpublished data) strains lacking a functional *ADE2 *gene (*ade2Δ-101*) were transformed with a circular fragment of chromosome III encoding *SUP11 *(Circle III/*SUP11*) [35] and incubated for 4 days on YPD at 30°C. *SUP11 *suppresses the *ade2Δ-101 *mutation resulting in the formation of white colonies. Loss of the CircleIII/*SUP11 *(an indicator of chromsome mis-segregation) was identified by the restoration of the *ade2Δ-101 *mutant phenotype causing the accumulation of P-ribosylaminoimidazole, a red colored pigment [41].

### Synthetic genetic array (SGA) analysis of *glc7-E101Q*

Two-independent SGA screens were performed in triplicate using the ordered deletion array and Virtek pinning robot system as previously described [12,13,71]. The sizes of the resulting colonies were measured from digital images of the plates. A comparative set of mutant measurements relative to wild type control measurements enabled t-statistics and p-values to be calculated [13]. Double mutants that showed significantly reduced colony sizes (*p *< 0.05) were scored as hits. Hits identified in the SGA screens were evaluated by a modified spot assay version of random spore analysis [13] using a *glc7-E101Q *strain that co-expressed *GLC7-res *(YNS100) (Additional File 3) and finally scored as synthetic sick/lethal (SSL) or rejected as false positive. Spores were initially grown for 2 days at 30°C on solid haploid selection medium [synthetic dextrose (SD) medium lacking histidine and arginine but containing canavanine: SD - His/Arg + canavanine]. *MAT*a spore progeny were transferred to solid 5-fluoro-orotic acid (5-FOA) medium [45] and incubated at 30°C for 2 days to eliminate cells expressing *GLC7-res*. Cells were inoculated to microtiter plates in 250 μl of liquid SD - His/Arg + canavanine for 2 days at 30°C. A Biomek FX robot (Beckman Coulter, Inc; CIAN robotics facility, McGill University) was used to perform serial dilutions and spotting to four different solid medium plates: SD/MSG (monosodium glutamic acid) - His/Arg + canavanine/nourseothricin, SD/MSG - His/Arg + canavanine/G418, SD/MSG - His/Arg +canavanine/nourseothricin/G418, and SD/MSG - His/Arg/Ura + canavanine. Colony growth was scored following incubation for 2 days at 30°C.

### Protein extraction, 1D and 2D-SDS-PAGE

All steps were performed at 4°C unless otherwise indicated. Log phase yeast cultures were harvested and washed in PGSK^+ ^buffer (PGSK^+^: 50 mM NaPO4, 50 mM NaCl, 5 mM KCl, 60 mM glucose, 4% CHAPS, 50 mM DTT, 5 μM yeast protease inhibitors [Sigma P8215], 5 μM PMSF, 5 μM ortho-vanadate, 5 μM NaF, and 5 mM β-glycerol phosphate) and pelleted by centrifugation. Cell pellets were suspended in 2 volumes of PGSK+, and an equal volume of zirconium beads (Biospec Products) added, and cells lysed by vortexing for 12–15 min. Extracts were cleared by centrifugation for 10 min at 13,000 × g. For 1D SDS-PAGE, extracts were re-suspended in an equal volume of 2× SDS sample buffer [72] and denatured at 95°C for 5 min. For 2D-SDS-PAGE and 2D-DiGE, extracts were treated with 50 μg/ml DNAse and RNAse (Worthington) for 15 minutes, precipitated with methanol/chloroform and re-suspended in labeling buffer (2 M thiourea, 7 M urea, 30 mM Tris, 4% CHAPS) at 25°C and the protein concentration determined (Biorad Protein Assay). For 2D-SDS-PAGE analysis of *GLC7-ProA *(YNS23) and *glc7-E101Q-ProA *(YNS27), 30 μg was loaded to 7 cm pH 4–7 Readystrip IPG strips (Biorad) and rehydration and focusing carried out according to the manufacturer's instructions.

### Immunoblotting

Immunoblotting was performed as previously described [65]. Briefly, proteins extracts prepared from *GLC7-ProA *(YNS23, YNS44) and *glc7-E101Q-ProA *(YNS26, YNS27) strains were separated on 1D or 2D-SDS-PAGE gels, transferred to PVDF membranes (Millipore) and probed with anti-ProA monoclonal antibodies (1:5000; clone SPA-27, Sigma). Actin was detected using the monoclonal antibodies (1:5000; clone C4, MP Biomedical). Protein/antibody complexes were detected using anti-mouse HRP-conjugated secondary antibodies (1:10,000; GE HealthCare) and ECL chemiluminescence (Pierce).

## Authors' contributions

ML, TN and NS constructed the strains, conducted the screens and ran data confirmation. JK, HP, MZ and MN helped in the data confirmation. PH, HB and CAM participated in the experimental design and data analysis and helped to draft the manuscript. ML, JV and GL conceived the study, analyzed the data and wrote the manuscript. All authors have read and approved the final manuscript.

## Supplementary Material

Additional file 1 PP1-mediated phosphate exchange. Click here for file

Additional file 2 Western blot analysis of Glc7-ProA and glc7-E101Q-ProA fusion proteins. Click here for file

Additional file 3 SGA and random spore analysis methods. Click here for file

Additional file 4 List of genes included in the SGA screens, found as hits in the screens and confirmed as *glc7-E101Q *SSL by random spore analysis. Click here for file

Additional file 5 List of *glc7-E101Q *SSL genes with and their attributes. Click here for file

Additional file 6 Distribution of high-level GO annotations for *glc7-E101Q *SSL genes. Click here for file
