# Supplementary material for: Genetic interaction network of the Saccharomyces cerevisiae type 1 phosphatase Glc7
Source: BMC Genomics. 2008 Jul 15;9:336. doi: 10.1186/1471-2164-9-336 (PMC2481269; doi:10.1186/1471-2164-9-336)
Supplement: Additional file 1 — PP1-mediated phosphate exchange. [file 1471-2164-9-336-S1.pdf]

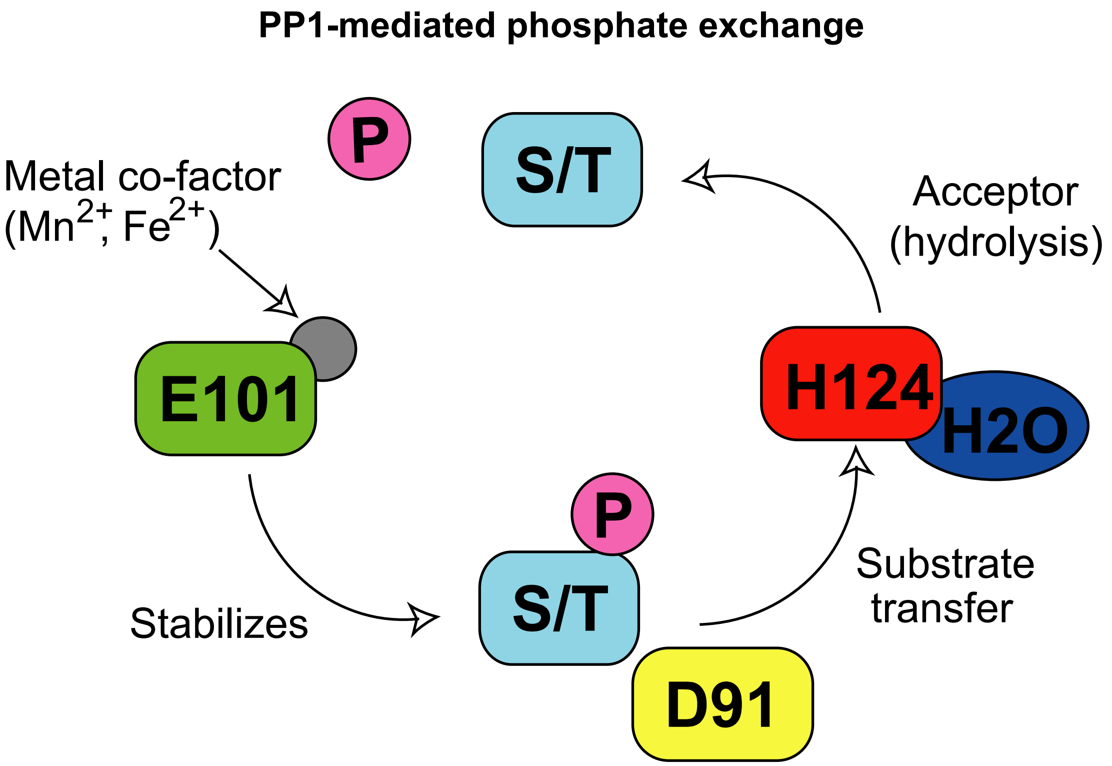

Additional File 1: PP1-mediated phosphate exchange. Phosphate exchange mediated by Glc7 is predicted to utilize D91 and H124 as phosphate transfer and phosphate acceptor sites, respectively. E101 is predicted to bind a metal cofactor (likely Mn2+ or Fe2+) that is required to accelerate phospho-transfer to H124.
