# Supplementary material for: Genetic interaction network of the Saccharomyces cerevisiae type 1 phosphatase Glc7
Source: BMC Genomics. 2008 Jul 15;9:336. doi: 10.1186/1471-2164-9-336 (PMC2481269; doi:10.1186/1471-2164-9-336)
Supplement: Additional file 3 — SGA and random spore analysis methods. [file 1471-2164-9-336-S3.pdf]

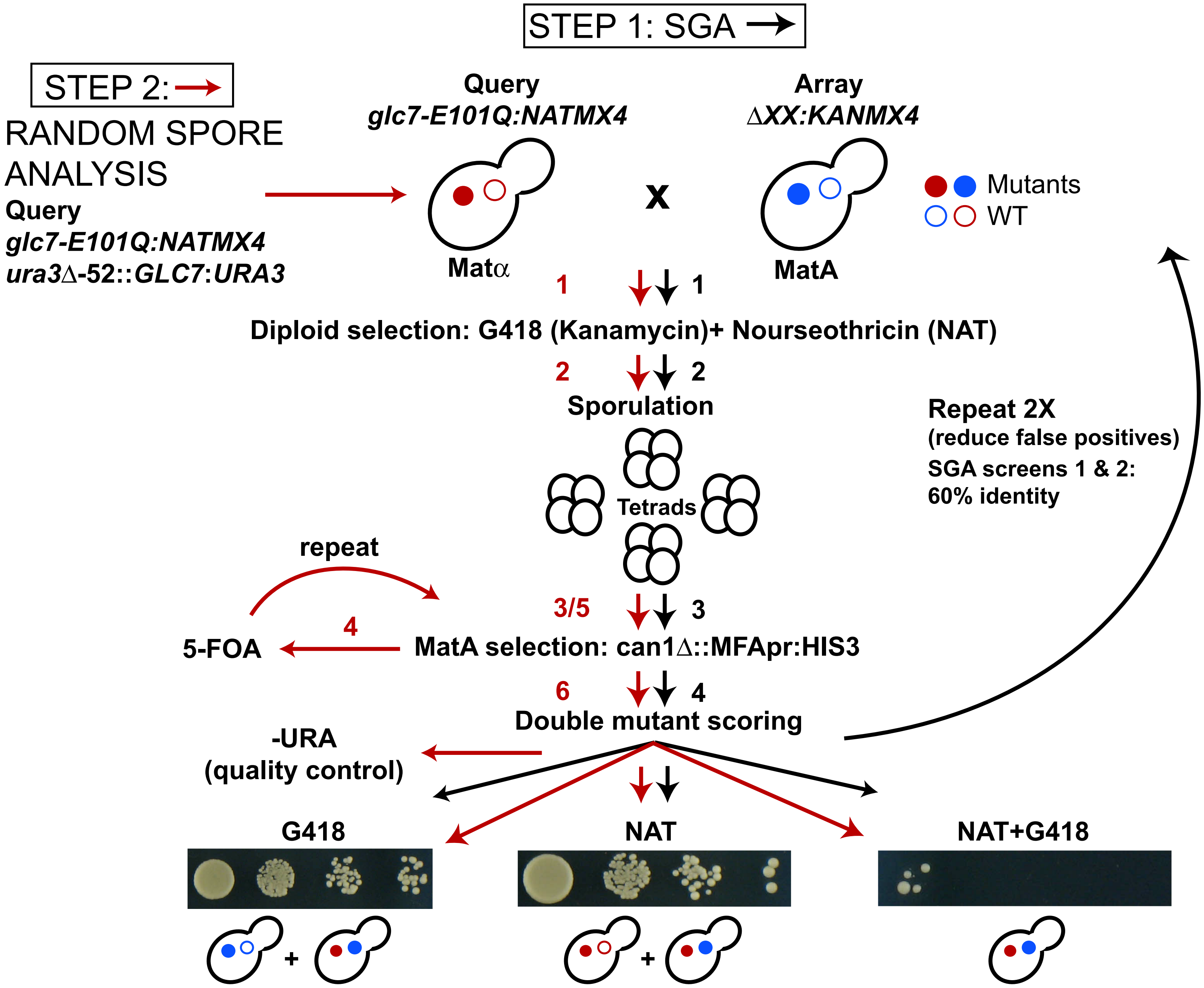

Additional File 3: SGA and random spore analysis methods. In the modified SGA confirmation scheme (red arrows) mutants found in the screen were mated with a *glc7-E101* strain bearing a wild type *GLC7* allele integrated at the *URA3* locus. After diploid selection (1), sporulation (2) and haploid *MATa* selection (3), 5-FOA resistant haploid *MATa* were selected (4,5). Finally, mutant scoring was made by spotting on G418 (both array and double mutant selection), Nourseothricin (NAT; both query and double mutant selection) and Nourseothricin + G418 (NAT+G418; double mutant selection).
