# Supplementary material for: Genetic interaction network of the Saccharomyces cerevisiae type 1 phosphatase Glc7
Source: BMC Genomics. 2008 Jul 15;9:336. doi: 10.1186/1471-2164-9-336 (PMC2481269; doi:10.1186/1471-2164-9-336)
Supplement: Additional file 6 — Distribution of high-level GO annotations for glc7-E101Q SSL genes. [file 1471-2164-9-336-S6.pdf]

GO_term	SSL	Array	*p*-value
amino acid and derivative metabolic process	5	167	0.947959854
anatomical structure morphogenesis	12	114	0.016883003
aromatic compound metabolic process	1	55	0.951053271
biological_process unknown	62	1362	0.941191102
carbohydrate metabolic process	4	130	0.92148041
cell budding	7	55	0.024969026
cell cycle	13	208	0.309786644
cell wall organization and biogenesis	5	177	0.963404856
cellular homeostasis	7	103	0.305149839
cofactor metabolic process	5	111	0.710292888
conjugation	6	92	0.362403478
cytokinesis	7	83	0.149562233
cytoskeleton organization and biogenesis	9	128	0.237727046
DNA metabolic process	15	224	0.207723892
generation of precursor metabolites and energy	3	124	0.964807876
lipid metabolic process	9	170	0.553244072
meiosis	7	107	0.339930159
membrane organization and biogenesis	9	139	0.317236539
nuclear organization and biogenesis	7	28	0.000492546
organelle organization and biogenesis	61	825	0.002837622
other	55	899	0.130687135
protein catabolic process	3	112	0.942432082
protein modification process	30	397	0.028374733
pseudohyphal growth	4	58	0.370643218
response to chemical stimulus	11	362	0.988340016
response to stress	25	392	0.18974824
ribosome biogenesis and assembly	10	123	0.116118353
RNA metabolic process	18	272	0.193299442
signal transduction	8	175	0.720213451
sporulation	4	112	0.854193796
transcription	7	103	0.305149839
translation	14	245	0.426838097
transport	38	701	0.470452968
vesicle-mediated transport	10	243	0.841386181
vitamin metabolic process	3	69	0.717900341

Additional File 6: Distribution of high-level GO annotations for *glc7-E101Q* SSL genes. The numbers of SSL and array genes whose biological process is annotated with a given GO term are indicated. The total number of SSL and array genes is 245 and 4617, respectively. Using a hypergeometric distribution model, the observed frequency for a given attribute in the SSL genes list was compared to that found for the set of array genes, and a *p*-value was calculated as follows:

$$p = 1 - \sum_{i=0}^{k-1} \frac{\binom{C}{i}\binom{G-C}{n-i}}{\binom{G}{n}}$$

, where *G* is the number of genes annotated (4617), *C* is the number of genes in the array having that attribute (Array), *n* is the number of SSL genes (245) of which *k* are known to possess the attribute (SSL).
