# Supplementary material for: Genetic interaction network of the Saccharomyces cerevisiae type 1 phosphatase Glc7
Source: BMC Genomics. 2008 Jul 15;9:336. doi: 10.1186/1471-2164-9-336 (PMC2481269; doi:10.1186/1471-2164-9-336)
Supplement: Additional file 2 — Western blot analysis of Glc7-ProA and glc7-E101Q-ProA fusion proteins. [file 1471-2164-9-336-S2.pdf]

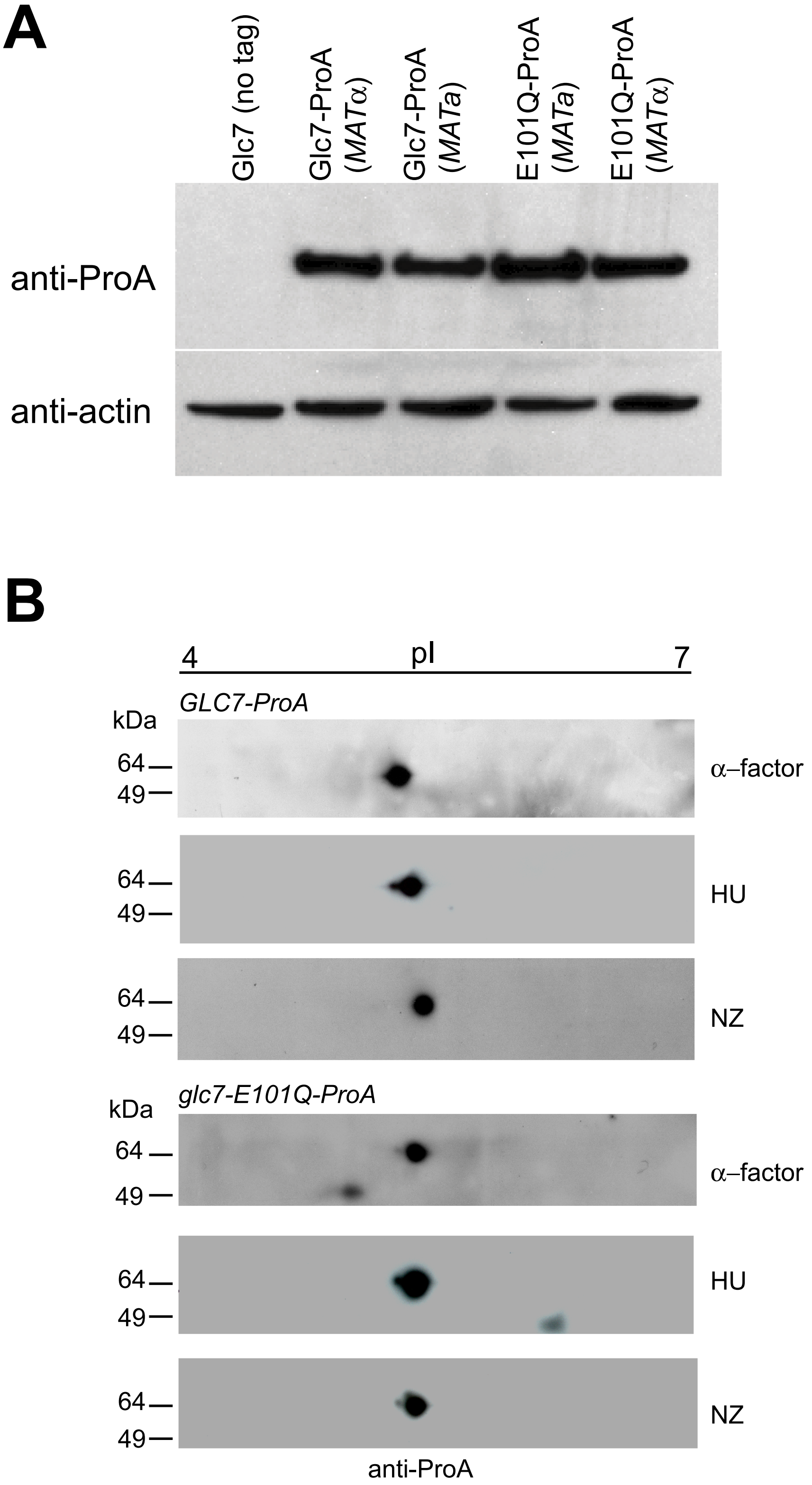

Additional File 2: Western blot analysis of Glc7-ProA and glc7-E101Q-ProA fusion proteins.
(A) Western blot for glc7-E101Q-ProA fusions compared to wild type (Glc7-ProA). Proteins from whole cell extracts (WCE) were separated by SDS-PAGE (50 μg/lane) and blotted with anti-ProA mAb. Protein load is shown by immunoreactivity using anti-actin antibodies (B) MATa cells expressing Glc7-ProA or glc7-E101Q-ProA were treated for 3 hours with 5 μg/ml α-factor (G1 arrest), 200mM hydroxyurea (S-phase arrest) or 20 μg/ml nocodozole (G2/M arrest) and WCE generated. 2D-SDS-PAGE was performed using pH 4-7cm Biorad ReadyStrip IPG strips (30 μg/strip) and blotted with anti-ProA mAb.
